# Embryonic expression of priapulid Wnt genes

**DOI:** 10.1007/s00427-019-00636-6

**Published:** 2019-07-04

**Authors:** Mattias Hogvall, Bruno C. Vellutini, José M. Martín-Durán, Andreas Hejnol, Graham E. Budd, Ralf Janssen

**Affiliations:** 1 Department of Earth Sciences, Palaeobiology, Uppsala University, Villavägen 16, Uppsala, Sweden; 2 Sars International Centre for Marine Molecular Biology, University of Bergen, Thormøhlensgate 55, 5006 Bergen, Norway; 3 Max Planck Institute of Molecular Cell Biology and Genetics, Pfotenhauerstraße 108, 01307 Dresden, Germany; 4 Present Address: School of Biological and Chemical Sciences, Queen Mary University of London, Mile End Road, London, E1 4NS UK

**Keywords:** Ecdysozoan ancestor, Wnt signalling, Evolution, Penis worm, Posterior elongation

## Abstract

Posterior elongation of the developing embryo is a common feature of animal development. One group of genes that is involved in posterior elongation is represented by the Wnt genes, secreted glycoprotein ligands that signal to specific receptors on neighbouring cells and thereby establish cell-to-cell communication. In segmented animals such as annelids and arthropods, Wnt signalling is also likely involved in segment border formation and regionalisation of the segments. Priapulids represent unsegmented worms that are distantly related to arthropods. Despite their interesting phylogenetic position and their importance for the understanding of ecdysozoan evolution, priapulids still represent a highly underinvestigated group of animals. Here, we study the embryonic expression patterns of the complete sets of Wnt genes in the priapulids *Priapulus caudatus* and *Halicryptus spinulosus*. We find that both priapulids possess a complete set of 12 Wnt genes. At least in *Priapulus*, most of these genes are expressed in and around the posterior-located blastopore and thus likely play a role in posterior elongation. Together with previous work on the expression of other genetic factors such as *caudal* and *even-skipped*, this suggests that posterior elongation in priapulids is under control of the same (or very similar) conserved gene regulatory network as in arthropods.

**Electronic supplementary material:**

The online version of this article (10.1007/s00427-019-00636-6) contains supplementary material, which is available to authorized users.

## Introduction

Wnt signalling is crucial for animal development, as it is involved in the regulation of numerous developmental processes such as cell proliferation and cell migration, organogenesis and pattern formation. Wnt genes encode secreted glycoprotein ligands that bind to various transmembrane receptors such as seven-pass Frizzled receptors and the receptor tyrosine kinases Ryk and Ror. Binding of Wnt(s) to their receptors induces intracellular gene cascades that lead (in the canonical Wnt pathway) to the release of beta-catenin which then regulates the transcription of Wnt target genes (reviewed in e.g. Logan and Nusse 2004, Croce and McClay 2008, Wiese et al. 2018). Wnt genes are subdivided into 13 classes, of which 12 are present in protostomian animals; the Wnt3-class was lost in the lineage leading to Protostomia (e.g. Kusserow et al. 2005; Cho et al. 2010; Janssen et al. 2010). One of the key functions of Wnt signalling is its general involvement in posterior growth in animals (e.g. Yazawa et al. 2009; McIntyre et al. 2013; Scimone et al. 2016; Kawai et al. 2016; Leclère et al. 2016). Hence, in “overtly segmented” animals, i.e. vertebrates, annelids and panarthropods, Wnt signalling is a key component of posterior segment addition (reviewed in Aulehla and Herrmann 2004, Murat et al. 2010, Cho et al. 2010, Janssen et al. 2010, Pruitt et al. 2014). While the specific gene regulatory networks (GRN) controlling posterior growth and segmentation can differ between different groups of animals, and in some cases even between closely related species of the same group, Wnt signalling seems to be always involved. However, the molecular interactions of Wnt signalling in posterior growth are far from being resolved. In many groups of animals, Wnt signalling appears to interact with other conserved posterior factors such as Even-skipped (Eve), Caudal (Cad) and Delta-Notch (Dl/N) signalling in the form of partially conserved GRNs (e.g. Chawengsaksophak et al. 2004; de Rosa et al. 2005; Shimizu et al. 2005; Chesebro et al. 2013; McGregor et al. 2009; Oberhofer et al. 2014). A fact that complicates the understanding of posterior Wnt signalling is that different combinations of Wnt genes (and other components such as Wnt receptors) are involved in posterior Wnt signalling where they play redundant and/or complementary function(s). In arthropods for example, several Wnt genes are frequently expressed in the posterior-located segment addition zone from where new segments are added to the growing embryo (e.g. Janssen et al. 2010; Hayden and Arthur 2014; Constantinou et al. 2016), and functional studies revealed that at least some of them (as well as other factors of Wnt signalling) likely fulfil combinatorial and/or redundant function(s) in posterior elongation and segmentation (e.g. Bolognesi et al. 2008; Beermann et al. 2011; Murat et al. 2010).

Another conserved function of Wnts is their role in morphological and molecular border formation (and maintenance) within the developing embryo. In the fly *Drosophila melanogaster*, other arthropods and even tardigrades and onychophorans, *wg* is likely involved in maintaining (para-) segmental boundaries and defining each segment’s polarity (Sanson 2001; Janssen et al. 2004; Gabriel and Goldstein 2007; Eriksson et al. 2009), and gene expression data on other Wnt genes suggest their involvement in intrasegmental patterning and segment border formation/maintenance (e.g. Janssen et al. 2010; Hogvall et al. 2014; Hayden and Arthur 2014; Constantinou et al. 2016).

**Fig. 1:**
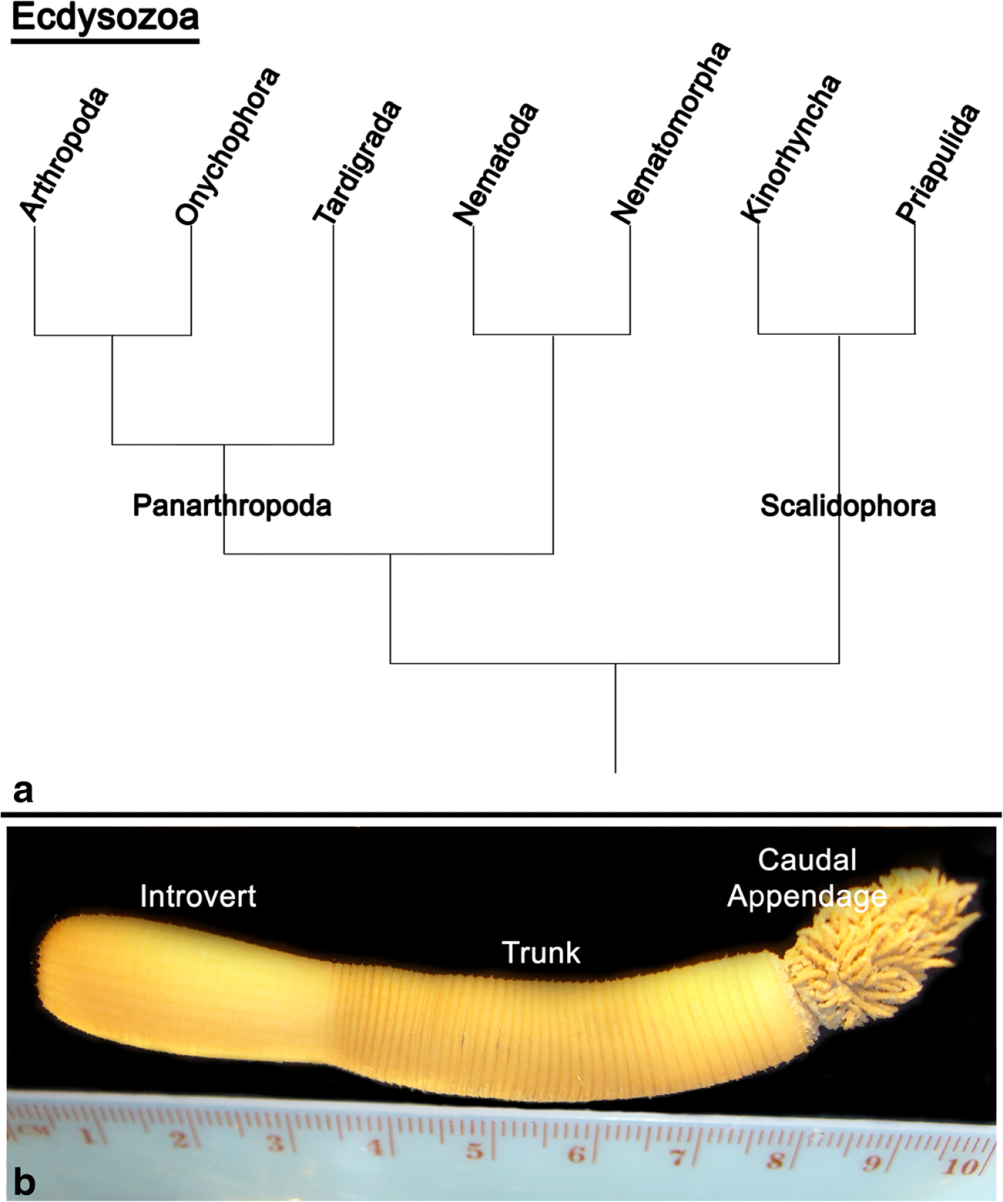
(**A**) Simplified cladogram representing the phylogenetic relationships of different ecdysozoan groups (after Campbell et al. 2011). (**B**) Adult specimen of the priapulid worm *Priapulus caudatus* next to a centimetre scale bar. Note subdivision of the adult body into the anterior introvert, the trunk and the posterior caudal appendage

Priapulids (penis worms) represent a group of unsegmented ecdysozoan animals closely related to kinorhynchs (mud dragons) and loriciferans (brush heads), which together comprise the Scalidophora (Schmidt-Rhaesa 1998, Nielsen 2012, Borner et al. 2014, reviewed in Giribet and Edgecombe 2017). Scalidophorans represent a group of ecdysozoans that may represent the sister group to all other ecdysozoans including arthropods and their close relatives (Campbell et al. 2011; Borner et al. 2014; Laumer et al. 2015) (Fig. 1(A)). However, note that ecdysozoan phylogeny is still not fully resolved (reviewed in Giribet and Edgecombe 2017).

Here, we investigate the Wnt gene complement, the stage-specific embryonic expression profiles and the embryonic expression patterns of the complete set of Wnt genes in the priapulid worm *Priapulus caudatus* (Fig. 1(B)). Our data reveal that several Wnt genes are expressed in specific posterior patterns, strongly suggesting a conserved role of Wnt genes in posterior growth in members of this group of ecdysozoan animals. However, none of the investigated Wnt genes is expressed in a pattern that could possibly represent a molecular remnant of body segmentation, or border formation in general.

## Methods

### Animal collection, fertilisation and embryo fixation

Sexually mature specimens of *Priapulus caudatus* were collected from Gullmarsfjorden (Fiskebäckskil, Sweden) in the area between Lysekil and Fiskebäckskil close to the Sven Lovén Centre for Marine Infrastructure. Mud was collected with a ring dredge from depths between approximately 30 and 60 m. Gonads of mature specimens were dissected and kept in filtered deep seawater (fDSW). The oocytes were then released by shaking the ovaries and after cleaning with fDSW; active sperm from several males was used for in vitro fertilisation. Fertilised eggs were kept in Petri dishes in filtered fDSWand incubated at a constant temperature of 10 °C. The eggs were washed daily with fresh fDSW to keep dishes free from overgrowth with bacteria, fungi and protozoans. Around 10 days after fertilisation, larvae hatched and these so-called hatching larvae then developed into the first lorica larvae after 1 week (Wennberg et al. 2009). Batches of embryos were collected of each developmental stage (defined as day after fertilisation (DAF)) for either RNA extraction or in situ hybridisation. These latter embryos were permeabilised prior to fixation with 0.05% thioglycolate, 0.01% pronase in fDSW for 45 min at 10 °C. After several washes in fDSW, the embryos were fixed in 4% paraformaldehyde in fDSWfor 1 h at room temperature, followed by several washes in phosphate-buffered saline with 0.1% Tween-20 (PBST). Samples fixed for gene expression studies were dehydrated in 50% methanol in PBST, washed once in 100% methanol and then stored in methanol at − 20 °C. Embryos used for RNA extraction and subsequent RT-PCRs were shock-frozen at − 80 °C and stored until RNA was extracted.

### Phylogenetic analysis

Reciprocal BLAST search using *Drosophila melanogaster wingless/Wnt1* (*wg/Wnt1*) against sequenced transcriptomes of the priapulids *Priapulus caudatus* (SRX507009) and *Halicryptus spinulosus* (SRX1343820) identified 12 Wnt-like genes per species. Amino acid sequences of the Wnt genes from *Priapulus*, *Halicryputs*, various arthropods, the onychophoran *Euperipatoides kanangrensis*, the annelid *Platynereis dumerilii* and of *Homo sapiens* were aligned using T-Coffee followed by manual editing in SeaView (Notredame et al. 2000; Gouy et al. 2010) with default parameters in MacVector v12.6.0 (MacVector, Inc., Cary, NC). The phylogenetic analysis was conducted using MrBayes (Huelsenbeck and Ronquist 2001). A fixed WAG amino acid substitution model with gamma-distributed rate variation across sites (with four rate categories), unconstrained exponential prior probability distribution on branch lengths and exponential prior for the gamma shape parameters for among-site rate variation were applied. Gene topology was calculated using 13,000,000 cycles for the Metropolis-Coupled Markov Chain Monte Carlo (MCMCMC) analysis (four chains; chain-heating temperature of 0.2). Markov chains were sampled every 200 cycles and default settings of 25% of samples were applied as burn-in. Clade support was calculated with posterior probabilities in MrBayes.

### Stage-specific RNA-seq analyses

Following the collection and fertilisation procedures above, we obtained bulk embryonic samples for a series of developmental stages that include oocytes and embryos at days 1, 3, 5, 7 and 9 post fertilisation. For this time course, we generated two biological replicates each coming from a common fertilisation using eggs from a single female individual and a sperm mix from different males. At least a thousand embryos were manually picked and transferred directly to RNAlater. We used TRIZOL (Invitrogen) to extract total RNA from each time point and sequenced single-end 50 base-pair reads of eleven samples using a single Illumina HiSeq2000 lane at the GeneCore (EMBL Genomics Core Facilities). One replicate of the 7-day sample failed at library preparation and was not included. We used kallisto (Bray et al. 2016) to quantify the transcript abundances by pseudoaligning the reads to a reference transcriptome (SRA Accession: SRX507009).

### Gene cloning and whole-mount in situ hybridisation

Total RNA from a mix of developmental stages of *Priapulus caudatus* embryos and larvae was extracted using TRIZOL (Invitrogen). All investigated gene fragments were amplified by means of RT-PCR from total RNA that was reverse transcribed into cDNA. Gene-specific primers were designed based on available sequence information. For all genes, nested PCRs were conducted with internal primers, using a first PCR as template (see Supplementary Table 1 for primer sequence information). Amplified gene fragments were cloned into the PCRII vector (Invitrogen) and sequenced on an ABI3730XL automatic sequencer (Macrogen, Seoul, South Korea). Identification numbers are summarised in Supplementary Table 2.

Single colorimetric in situ hybridisation was performed as described in Martín-Durán et al. (2012).

### Data documentation

Prior to being photographed, embryos were incubated in 70% glycerol and mounted on glass slides under a thin glass cover. A Leica DFC550 digital camera mounted onto a Leica Leitz DMRXE dissection microscope was used. Whenever needed, contrast and brightness were adjusted using the image-processing software Adobe Photoshop CS6 for Apple Macintosh (Adobe Systems Inc.).

## Results

### Sequence analysis

**Fig. 2:**
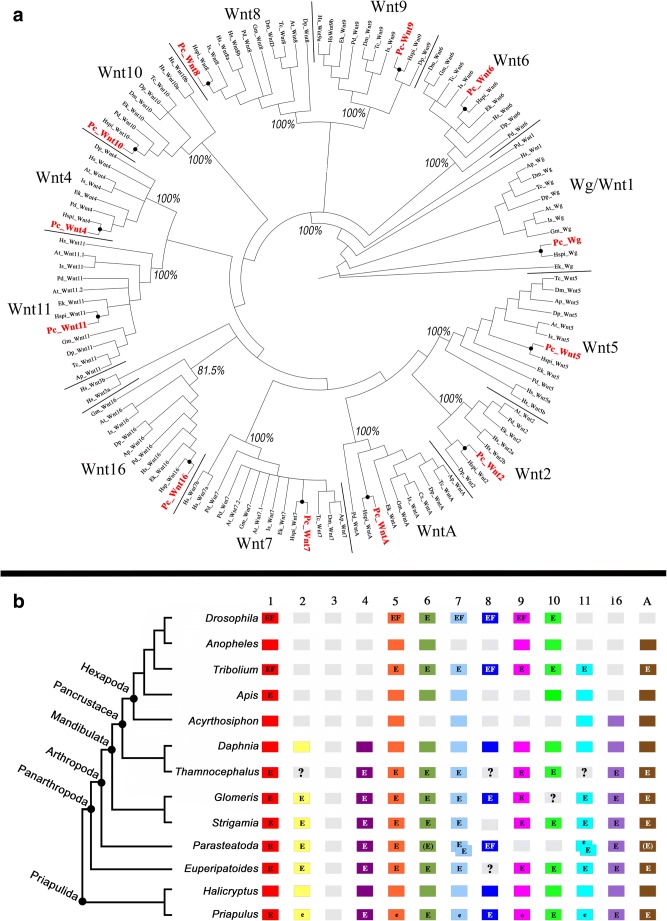
(**A**) Phylogenetic analysis of Wnt genes. Bayesian analysis of Wnt amino acid sequences. Support of each node is given as posterior probability. Included species are as follows: *Achaearanea* (syn. *Parasteatoda*) *tepidariorum* (At), *Acyrthosiphon pisum* (Ap), *Cupiennius salei* (Cs), *Daphnia pulex* (Dp), *Drosophila melanogaster* (Dm), *Euperipatoides kanangrensis* (Ek), *Glomeris marginata* (Gm), *Halicryptus spinulosus* (Hspi), *Homo sapiens* (Hs), *Ixodes scapularis* (Is), *Platynereis dumerilii* (Pd), *Priapulus caudatus* (Pc) and *Tribolium castaneum* (Tc). (**B**) Wnt gene complement in arthropods, an onychophoran and priapulids. Grey boxes indicate lost Wnt subfamilies. Question marks in grey boxes indicate Wnts that have not been found in sequenced embryonic transcriptomes. Duplicated Wnts are represented by overlapping boxes. (E) Unpublished embryonic expression pattern (R. Janssen); E, published embryonic expression pattern; e, embryonic expression has been investigated but specific expression patterns were not detected; F, functional data are available

Phylogenetic analysis confirms that the identified genes fall into the expected 12 classes of Wnt genes (Fig. 2(A)), showing that priapulids possess the full set of Wnt genes reported for protostomes (e.g. Janssen et al. 2010) (Fig. 2(B)). All priapulid Wnt genes (except Wnt16) cluster with absolute support (100%) with confirmed Wnt orthologs from arthropods, an onychophoran (*Euperipatoides kanangrensis*), an annelid (*Platynereis dumerilii*) and human (*Homo sapiens*). Support for a monophyletic clade containing all Wnt16 orthologs is sufficiently high (81.5%) to suggest that this represents a monophyletic group as well.

### Expression analysis based on stage-specific quantitative RNA sequencing

**Fig. 3:**
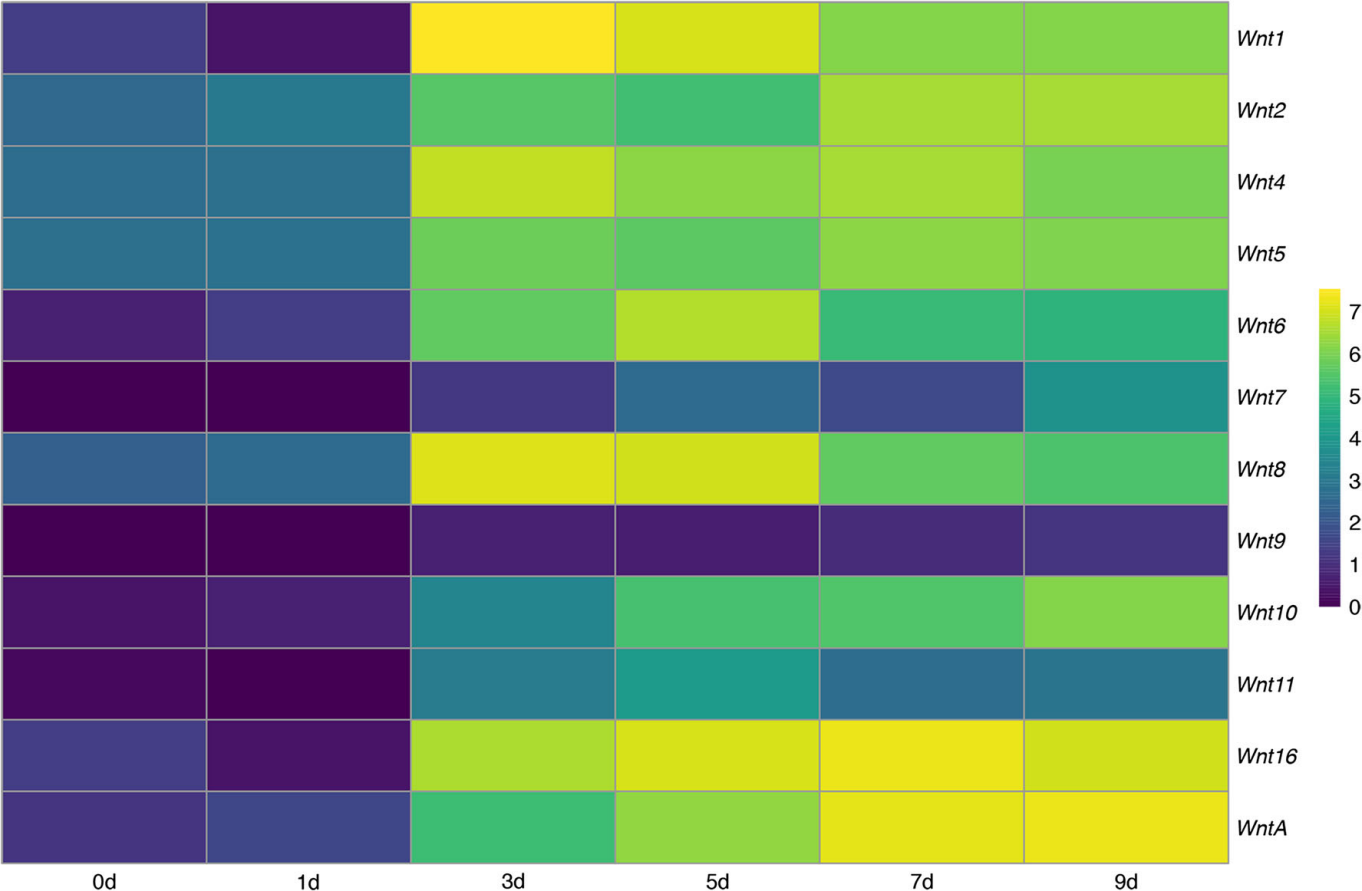
Expression of Wnt genes during *P. caudatus* embryogenesis. The estimated counts were averaged between replicates and summed between putative isoforms, and log transformed to visualise the gene-level abundances across developmental stages

We used stage-specific RNA-seq data from a time course including six different developmental stages (oocytes, 1 day after fertilisation (DAF), 3DAF, 5DAF, 7DAF, and 9DAF) to analyse the quantitative expression profile of *Priapulus* Wnt genes over time. Our data revealed that none of the *Wnt genes* is maternally expressed, and none of the Wnt genes is active early during development (1DAF) (Fig. 3). During gastrulation (3 DAF), however, all Wnt genes except for *Wnt7*, *Wnt9*, *Wnt10* and *Wnt11* are transcribed at a significant level (Fig. 3). At 5DAF, *Wnt10* appears to be transcribed (albeit at a significantly lower level than the other active Wnt genes). All nine active Wnt genes, Wnts 1, 2, 4, 5, 6, 8, 10, 16 and A, appear to be expressed at all subsequent developmental stages until the pre-hatching stage.

### Expression of *Priapulus caudatus* Wnt genes

All priapulid Wnt genes for which we obtained reliable whole-mount in situ hybridisation expression data are detected at the posterior pole of the developing embryo, close to, in or around the blastopore (cf. expression of *wg/Wnt1* in the blastopore (Martín-Durán and Hejnol 2015)). We obtained data for late gastrula-stage embryos and so-called introvertula-stage embryos. The latter embryos are characterised by the formation of a groove that separates the posterior trunk from the anterior introvert (proboscis). Note that some embryos are slightly malformed as a result of the fixation procedure.

**Fig. 4:**
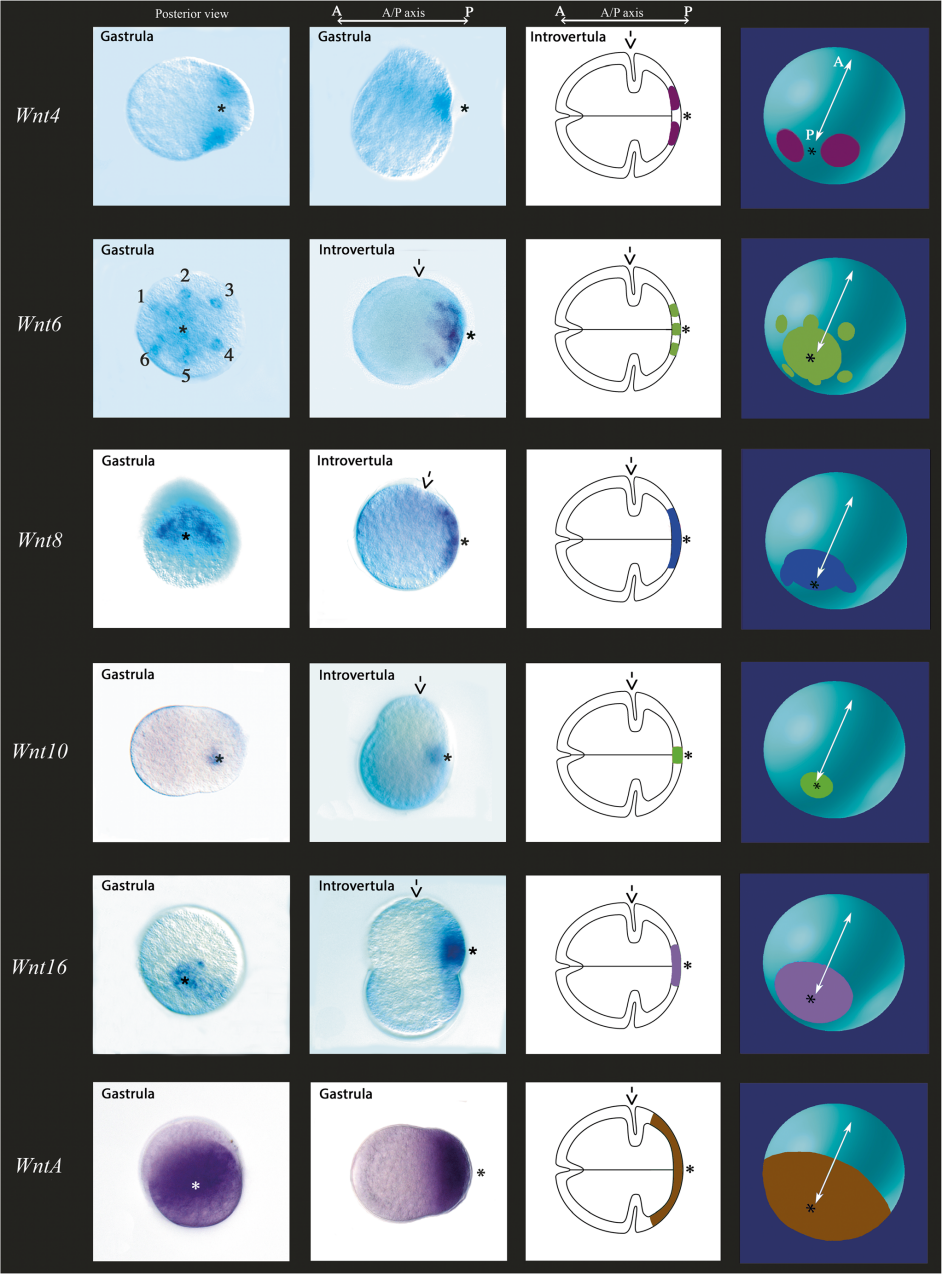
Embryonic expression patterns of *Priapulus caudatus* Wnt genes. In all panels, asterisks mark the posterior pole of the embryo. The first column shows embryos in posterior view (A, E, I, M, Q, U); the second column shows embryos in lateral view (B, F, J, N, R, V); the third column shows schematic expression in lateral view (cf. second column); the fourth column shows schematic expression in posterior–lateral view (the white arrows indicate the AP axis). Roman numerals in panel E indicate six dot-like expression domains surrounding the posterior pole of the embryo. In all data panels, the developmental stage is indicated. The arrows in panels showing the introvertula stage in lateral viewpoint to the groove separating the introvert from the trunk

*Wnt4* is expressed in the form of two domains, each on either side of the blastoporal region (Fig. 4(A, B)). Since determination of orientation of the embryo with respect to the left-right (LR) and dorsal-ventral (DV) axes is not possible due to the lack of morphological markers, we cannot decipher whether the expression domains are lateral to either side of the blastopore, or ventral and dorsal to the blastopore (although we assume lateral expression rather than dorsal and ventral domains).

*Wnt6* is expressed in a very specific ectodermal pattern in the posterior of the embryo. Expression is in and around the blastopore. Most significant, however, are the six dot-like expression domains surrounding the blastopore at the edge of a broad region of expression (Fig. 4(C, D)). This expression is similar to that of *FGF8/17/18* in the anterior of the developing embryo (Martín-Durán and Hejnol 2015) and may be correlated with the development of the short and/or long retractor muscles.

*Wnt8* is expressed in the form of a single strong posterior domain (Fig. 4(E, F)). However, this domain is restricted to one side of the blastopore opening. Again, owing to the lack of morphological landmarks, we cannot determine if this is dorsal or ventral (we do not assume left-right asymmetric expression).

*Wnt10* is expressed in the form of a small domain in the blastopore which is smaller than that of the other posteriorly expressed Wnt genes and is restricted to the blastoporal opening (Fig. 4(G, H)).

Expression of *Wnt16* is surrounding the blastopore in the form of a solid circular and broad domain (Fig. 4(I, J)).

*WntA* is expressed in the posterior half of the embryo, and thus in a much greater posterior domain as the other Wnt genes (Fig. 4(K, L)). The expression of *WntA* appears to be stronger in more posterior tissue, while expression towards its anterior border of (detectable) expression is weaker, possibly forming a short-range posterior to anterior gradient (Fig. 4(L)).

## Discussion

### Restricted expression of Wnt genes early during priapulid development

Of the 12 priapulid Wnt genes identified in sequenced transcriptomes, nine are expressed during embryonic development until 9 days after fertilisation (the first larval stage is reached at approximately 10 days after fertilisation (Wennberg et al. 2008)). Of these nine expressed Wnt genes (see Fig. 3), we retrieved reliable whole-mount in situ expression data for seven genes. The only Wnt genes that show expression in the stage-specific RNA-seq time course, and for which we could not obtain any in situ hybridisation signal, are *Wnt2* and *Wnt5*. One explanation could be that these genes are ubiquitously expressed at a low level. In such a case, it would be difficult to distinguish their expression pattern from background signal, a common problem in *Priapulus* in situ hybridisation experiments. If these genes are ubiquitously expressed, then the level of expression per cell must be low (cf. level of RNA-seq. signal (Fig. 3)).

### Expression of priapulid Wnt genes suggests an evolutionary conserved function of Wnt signalling in posterior patterning and posterior elongation

Wnt gene signalling appears to be a conserved component of posterior development and posterior elongation as reported from a wide range of metazoans.

Already in sponges like the demosponges *Amphimedon* and *Halisarca*, the expression of Wnt genes in posterior tissues indicates a potential role in the establishment of the AP axis during development (Adamska et al. 2007, 2010; Leininger et al. 2014; Borisenko et al. 2016). Similarly, Wnt signalling could be involved in oral/aboral patterning in Ctenophora (Jager et al. 2013, but see Pang et al. 2010). In cnidarians, Wnt signalling is involved in gastrulation and AP axis patterning (e.g. Hobmayer et al. 2000; Kusserow et al. 2005; Lee et al. 2006; Lengfeld et al. 2009). The function of Wnt signalling in posterior elongation and AP axis patterning is well documented in deuterostomes such as vertebrates (e.g. Schier and Talbot 2005) and expression pattern analysis suggests that Wnt signalling is also involved in these processes in other deuterostomes (e.g. Onai et al. 2009; Kawai et al. 2016; Darras et al. 2018). Although there are not much data on lophotrochozoan (spriralian) protostomes, at least in annelids Wnt genes appear to be involved in segmentation and posterior elongation as well (e.g. Janssen et al. 2010; Cho et al. 2010; Gazave et al. 2013).

In ecdysozoans, the other group of protostomian animals, data on Wnt signalling are mainly restricted to arthropods. Here, Wnt signalling and the posterior factors *caudal* (*cad*) and *even-skipped* (*eve*) interact in gene regulatory networks controlling segment addition and posterior elongation (e.g. Copf et al. 2004, Miyawaki et al. 2004, Shinmyo et al. 2005, Angelini and Kaufman 2005, Bolognesi et al. 2008, McGregor et al. 2008, 2009, Beermann et al. 2011, Chesebro et al. 2013, El-Sherif et al. 2014, Oberhofer et al. 2014, Hayden et al. 2015, Schönauer et al. 2016, Auman et al. 2017, reviewed in Williams and Nagy 2017). Gene expression data in onychophorans suggest that these genes may play a conserved role during onychophoran posterior elongation as well (Eriksson et al. 2009; Janssen and Budd 2013; Hogvall et al. 2014).

Data from non-panarthropod ecdysozoans, however, are scarce, except for the model nematode *Caeorhabditis elegans*. This species only possesses five Wnt genes (*mom-2*, *lin-44*, *egl-20*, *cwn-1* and *cwn-2*), most of which are expressed and function in posterior structures of the developing larva, or are active in a posterior organisation centre of the embryo that establishes anterior–posterior polarity (e.g. Bischoff and Schnabel 2006; Sawa and Korswagen 2013). The *Caenorhabditis caudal* ortholog *pal-1* is involved in posterior development (Edgar et al. 2001), and so is *vab-7*, an *even-skipped* (*eve*) ortholog (Pocock et al. 2004). Altogether, data on *cad*, *eve* and Wnt orthologs imply some sort of conserved function in posterior development in the nematode, at least in comparison with arthropods and onychophorans.

Data on Wnt genes and other factors possibly associated with posterior elongation are not available from other nematodes, nematomorphs or scalidophorans (kinorhynchs, loriciferans and priapulids), except for the expression data on *Priapulus wg/Wnt1*, *cad* and *eve* (Martín-Durán et al. 2012; Martín-Durán and Hejnol 2015), and data provided in this study.

Our data demonstrate that several Wnt genes are transcribed in, around or in close proximity to the posteriorly located blastopore. The only Wnt gene with a significantly different expression pattern, *WntA* is expressed in the complete posterior half of the embryo. These data, together with the previously published expression data on *wg/Wnt1*, *eve* and *cad*, strongly suggest a role of Wnt genes (and Wnt signalling) in posterior patterning and posterior elongation in priapulids, likely as part of a similar gene regulatory network as demonstrated for arthropods.

### Wnts and border formation

Wnt signalling appears to play an important role in intrasegmental patterning in segmented animals such as arthropods, onychophorans and annelids (e.g. Janssen et al. 2010; Hogvall et al. 2014). The most famous Wnt gene, *wingless* (*wg/Wnt1*) is a key factor in maintaining parasegmental boundaries in concert with other segment-polarity genes (SPGs) in *Drosophila* (reviewed in Sanson 2001). Similar segmentally reiterated expression patterns of *wg/Wnt1* orthologs and other SPGs in members in all arthropod clades, onychophorans and annelids suggest that the function of *wg/Wnt1* in border formation dates back to the last common ancestor of protostomes and deuterostomes (Damen 2002; Prud’homme et al. 2003; Janssen et al. 2004; Eriksson et al. 2009; Dray et al. 2010; Janssen and Budd 2013) and may thus lend support for a segmented last common ancestor (Balavoine and Adoutte 2003; Balavoine 2014). Although relatively little is known about the function of other Wnt genes in intrasegmental patterning and/or border formation, strikingly many Wnt genes are expressed in transverse segmental stripes in and around the (para) segmental boundaries in panarthropods and annelids (Janssen et al. 2010; Janssen and Posnien 2014; Hogvall et al. 2014; Hayden and Arthur 2014; Pruitt et al. 2014; Constantinou et al. 2016). These patterns suggest that Wnt genes may also be involved in the patterning of body units (segments) along the anterior–posterior (AP) body axis and thus border formation (either morphological borders such as segmental boundaries, or molecular borders such as the parasegment boundaries). Further, a role of Wnt genes in border formation in non-segmented animals has been recently shown for brachiopod larvae (Vellutini and Hejnol 2016).

Priapulids are not considered “segmented” animals as there are no segmentally repeated body structures, or morphologically visible boundaries except for the boundary between the anterior introvert and the posterior trunk. This boundary is established early during development, as the developing introvert is characterised by much smaller cells than the trunk which is formed from larger cells (Wennberg et al. 2008).

We found that none of the priapulid Wnt genes appears to be expressed at the border between introvert and trunk, at least not in the stages for which in situ hybridisation works in this species. It is possible that formation and maintenance of this morphological border do not require Wnt signalling. Alternatively, Wnt signalling may not be recognised owing to a low level of expression, or Wnt genes are expressed but at different developmental stages for which in situ hybridisation does not work. Additionally, we did not detect any sign of expression that would suggest that the ancestor of priapulids was segmented, such as cryptic expression of Wnt genes in reiterated patterns (transverse stripes) along the AP body axis; a lack that counts against the common origin of segmentation in annelids (Lophotrochozoa) and panarthropods (Ecdysozoa).

## Electronic supplementary material

Supplementary Figure 1 – RNAseq Raw Data (PNG 51 kb)

High-resolution image (TIF 1980 kb)

Supplementary Table 1 – Primer List (DOCX 15 kb)

Supplementary Table 2 – Accession numbers (DOCX 14 kb)
