# Supplementary figures and images for: Embryonic expression of priapulid Wnt genes
Source: Dev Genes Evol. 2019 Jul 4;229(4):125–35. doi: 10.1007/s00427-019-00636-6 (PMC6647475; doi:10.1007/s00427-019-00636-6)

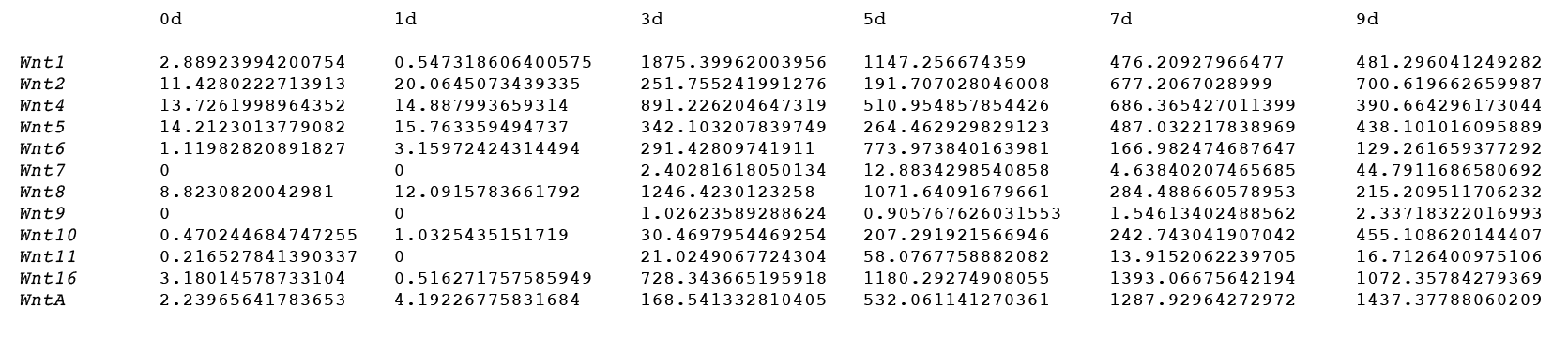

Supplement: Supplementary file 1 — – RNAseq Raw Data (PNG 51 kb) [file 427_2019_636_Fig5_ESM.png]

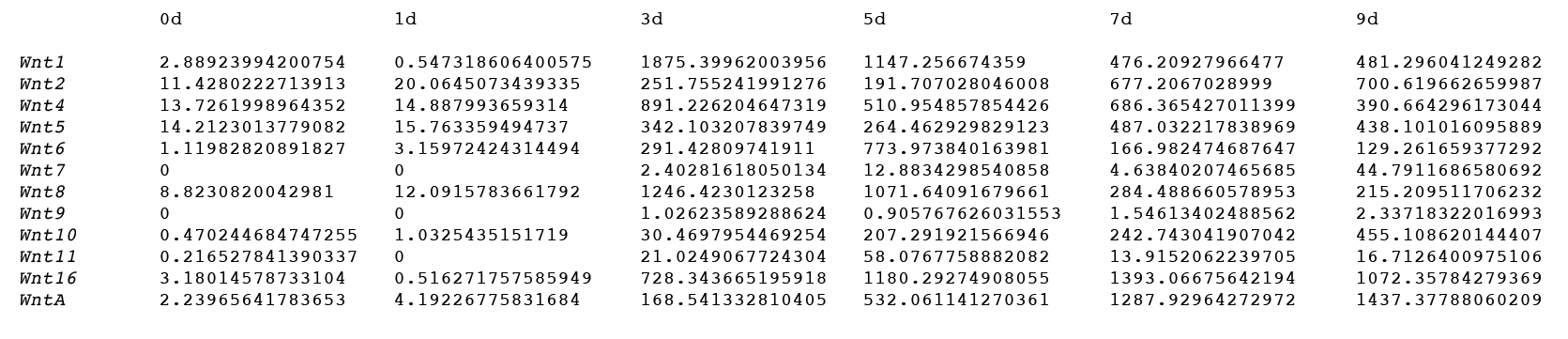

Supplement: Supplementary file 2 — High-resolution image (TIF 1980 kb) [file 427_2019_636_MOESM1_ESM.tif]
