# Supplementary material for: Embryonic expression of priapulid Wnt genes
Source: Dev Genes Evol. 2019 Jul 4;229(4):125–35. doi: 10.1007/s00427-019-00636-6 (PMC6647475; doi:10.1007/s00427-019-00636-6)
Supplement: Supplementary file 3 — – Primer List (DOCX 15 kb) [file 427_2019_636_MOESM2_ESM.docx]

Primer list:

| Gene | Forward 1 | Forward 2 | Reverse 1 | Reverse 2 |
| --- | --- | --- | --- | --- |
| *Wnt2* | GTGGTTTCTGAGTCAG | GGTGTCCATAGGAGAA | GACGAAAATCTGAGGG | CATCACTTCTTCCGAC |
| *Wnt4* | GTCCATAGAATGCGTG | CTCGTGGGCTTTATTG | CGGTTGTATAGTTGGG | GTAAGTTAAGGCCTGC |
| *Wnt5* | AAGAGAGTGGGAGATC | CGTATATATCGGTGGC | TCTCAACTCATCCTCC | GCTAGTATCGGGAGTT |
| *Wnt6* | CCCAGAGCAGTTTTAG | CGACTATACCAACGAC | GCATACATAGACTGCC | GCCAGAGATTCACTTG |
| *Wnt7* | GTCGCACACAGACTA | TGGACCTACTGCTGT | TTCGTCCCCATCGAA | GTAGTTGGGCGACTT |
| *Wnt8* | ACAGACAGACAGAGAG | GATAGAGCGACAGGTA | CATGAGTGCAGAGAAG | CATAAACACATCCGGG |
| *Wnt9* | TTCAAACTGAGCAGCAAACA | TAAATGGCATGGCGGAAACA | TGATGGATTGCATAGATACC | CCTAACGTGAATCGCATCTT |
| *Wnt10* | GATTCCACGACTTCTC | TCTCAGAACTTCTCCC | ACCTTCTCTGTACCAG | GAAAGATAGAGCACCG |
| *Wnt11* | CGTATCTGTGATAGCG | GGACAAACCAGGAATG | GTCTATTCTTGCAGCC | CTCACAGACACTGGT |
| *Wnt16* | GGATAGACACGAACAC | TCCCGGAGTACCTATA | GCTATACACGGGTAGA | CACGATGATATCTCGG |
| *WntA* | CAAGCTCGATAATCCC | CGTCAGTGTGATCTAC | GCTCATACGGTCATAC | CGTAAGTACGTGAGAC |
