# Supplementary material for: Embryonic expression of priapulid Wnt genes
Source: Dev Genes Evol. 2019 Jul 4;229(4):125–35. doi: 10.1007/s00427-019-00636-6 (PMC6647475; doi:10.1007/s00427-019-00636-6)
Supplement: Supplementary file 4 — – Accession numbers (DOCX 14 kb) [file 427_2019_636_MOESM3_ESM.docx]

Gene Fragment Identification Numbers:

| Gene | Identification Number |
| --- | --- |
| *Wnt2* | LR025119 |
| *Wnt4* | LR025120 |
| *Wnt5* | LR025121 |
| *Wnt6* | LR025122 |
| *Wnt7* | LR025123 |
| *Wnt8* | LR025124 |
| *Wnt9* | LR025125 |
| *Wnt10* | LR025126 |
| *Wnt11* | LR025127 |
| *Wnt16* | LR025128 |
| *WntA* | LR025129 |
